# Evaluation of Natalizumab Pharmacokinetics and Pharmacodynamics: Toward Individualized Doses

**DOI:** 10.3389/fneur.2021.716548

**Published:** 2021-10-07

**Authors:** Jose M. Serra López-Matencio, Yaiza Pérez García, Virginia Meca-Lallana, Raquel Juárez-Sánchez, Angeles Ursa, Lorena Vega-Piris, Dora Pascual-Salcedo, Annick de Vries, Theo Rispens, Cecilia Muñoz-Calleja

**Affiliations:** ^1^Servicio de Farmacia, Hospital Universitario de La Princesa, Madrid, Spain; ^2^Servicio de Inmunología, Hospital de La Princesa, Madrid, Spain; ^3^Servicio de Neurologia, Hospital Universitario de La Princesa, Madrid, Spain; ^4^Fundación Biomédica, Hospital de La Princesa, Madrid, Spain; ^5^Department of Immunopathology, Sanquin Research and Landsteiner Laboratory, Amsterdam University Medical Centre, University of Amsterdam, Amsterdam, Netherlands; ^6^School of Medicine, Universidad Autónoma de Madrid, Madrid, Spain

**Keywords:** natalizumab, pharmacokinetics, pharmacodynamics, multiple sclerosis, α4-integrin, dose scheme, efficacy, progressive multifocal leukoencephalopathy PML

## Abstract

**Background:** Plasma concentration of natalizumab falls above the therapeutic threshold in many patients who, therefore, receive more natalizumab than necessary and have higher risk of progressive multifocal leukoencephalopathy.

**Objective:** To assess in a single study the individual and treatment characteristics that influence the pharmacokinetics and pharmacodynamics of natalizumab in multiple sclerosis (MS) patients in the real-world practice.

**Methods:** Prospective observational study to analyse the impact of body weight, height, body surface area, body mass index, gender, age, treatment duration, and dosage scheme on natalizumab concentrations and the occupancy of α4-integrin receptor (RO) by natalizumab.

**Results:** Natalizumab concentrations ranged from 0.72 to 67 μg/ml, and RO from 26 to 100%. Body mass index inversely associated with natalizumab concentration (beta = −1.78; *p* ≤ 0.001), as it did body weight (beta = −0.34; *p* = 0.001), but not height, body surface area, age or gender Extended vs. standard dose scheme, but not treatment duration, was inversely associated with natalizumab concentration (beta = −7.92; *p* = 0.016). Similar to natalizumab concentration, body mass index (beta = −1.39; *p* = 0.001) and weight (beta = −0.31; *p* = 0.001) inversely impacted RO. Finally, there was a strong direct linear correlation between serum concentrations and RO until 9 μg/ml (rho = 0.71; *p* = 0.003). Nevertheless, most patients had higher concentrations of natalizumab resulting in the saturation of the integrin.

**Conclusions:** Body mass index and dosing interval are the main variables found to influence the pharmacology of natalizumab. Plasma concentration of natalizumab and/or RO are wide variable among patients and should be routinely measured to personalize treatment and, therefore, avoid either over and underdosing.

## Introduction

Natalizumab is a recombinant humanized anti-α4-integrin antibody against the α subunit (CD49d) of α4 integrins [α4β1 (VLA-4) and α4β7], that prevents the extravasation of inflammatory leukocytes across the blood-brain barrier into the central nervous system (1, 2). Natalizumab is currently indicated as a single disease modifying therapy in highly active relapsing remitting multiple sclerosis (RRMS) (3–5). The drug reduces multiple sclerosis relapses very effectively; nevertheless, it is associated with progressive multifocal leukoencephalopathy (PML), a potentially fatal complication caused by the reactivation of latent John Cunningham virus (JCV). Established risk factors for PML include: the level of anti JCV antibodies in serum as assessed by an anti-JCV antibody index; the use of immunosuppressant therapy before natalizumab initiation; and the duration of natalizumab treatment (6, 7).

The mechanisms underlying the development of PML associated with natalizumab are not completely understood but in all likelihood include, among others, the inhibition of the trafficking of immune cells like antigen presenting cells and anti-viral Th1 lymphocytes to the central nervous system (8, 9), therefore hampering the elimination of the JVC. The magnitude of this extravasation blockade is almost certainly related to the degree of saturation of the α4-integrin. Since demonstrating the presence of a concentration-dependent increase of α4-integrin saturation (10, 11), the PML risk seems to be linked to natalizumab serum concentrations (12–14).

Natalizumab is approved at a fixed dose of 300 mg IV every 4 weeks for the treatment of RRMS in adult patients, allowing natalizumab concentrations to be maintained at levels which ensure continuous maximal α4β1 integrin receptor saturation (15). However, many monoclonal antibodies are dosed on an individual basis (16, 17).

Whereas in the AFFIRM trial the average concentration on the steady state ranged between 23 and 29 μg/ml (11). It is known that plasma concentrations of natalizumab between 1 and 2 μg/ml are enough for most patients to reach saturation of α4-integrin [>80% of receptor occupancy (RO)]. Also that receptor desaturation (saturation <50%) only happens when natalizumab serum concentrations fall below 1 μg/mL (18). This suggests that most patients are overdosed by following the approved guidelines. In addition, considerable variation in natalizumab levels was found among patients in several studies (5, 10, 11, 19) despite administering the same dosage. As a consequence, many clinicians throughout the world now utilize alternative dosing schedules, mainly the extended interval dose (EID), to the standard interval dose (SID) (20–22).

We believe that optimizing natalizumab doses for individual patients is necessary to avoid either relapses or side-effects of the drug. To achieve this, it is necessary to know the impact of different parameters, including: body weight, height, body surface area, body mass index (BMI), age, gender, treatment duration and dosing intervals which influence the pharmacology of natalizumab. However, very few studies (19) have considered, in the real-world practice, both the personal and treatment characteristics altogether in the pharmacokinetics (PK) and pharmacodynamics (PD) of this therapeutic antibody.

## Materials and Methods

### Patients and Study Design

This study is a prospective, observational, nonrandomized, open-label study performed at La Princesa Hospital (Madrid, Spain). For the study were enrolled 32 patients receiving natalizumab for relapsing into various forms of RRMS between 2014 and 2019. The eligibility criteria were: a diagnosis of RRMS according to the applicable panel criteria (23), age of 18 years or older and receiving natalizumab treatment with a minimum of six consecutive infusions. Exclusion criteria were: patients who did not give their informed consent in writing to take part in the study, patients who did not follow the treatment and patients who did not meet the above criteria.

Blood samples were collected immediately prior to the next natalizumab infusion. For some analyses, patients were divided into two groups based on the time interval since the previous natalizumab infusion: SID (26–33 days) or EID (34–41 days). These ranges were chosen after considering the bibliography, in which an interval of 3.5–4.5 weeks was considered standard, and an interval of more than 5 weeks was considered as extended (19–21). Body surface area was calculated with the Du Bois formula (24) and BMI was calculated as body-weight/height^2^ (25). At least two serum samples and a maximum of four were drawn from each patient with a difference between samples of 1–5 months. Twenty three patients contributed with two samples, eight with three and one with four.

All samples were obtained at least 7 months after the first dose of natalizumab, therefore the drug had reached the steady state in all patients (10, 26). The longest time from the beginning of the treatment to the extraction of the peripheral blood samples was 54 months. In the case of SID and EID, the interval between sample collection and start of treatment is comparable (30.8 months for SID and 35.61 for EID) according to a two-sample *t*-test with equal variances, *p*-value = 0.1789. In total, 74 samples were collected: 30 corresponded to the SID group (40.54%), and 44 to the EID one (59.45%).

The study was classified as a “Postauthorization-Observational Study with drugs” by the Spanish Drug Agency and approved by the ethics committee of La Princesa Hospital (2634A; AMB-NAT-2015-01) and written, informed consent was provided by all research participants. Expanded disability scale score (EDSS), annualized relapse rate (ARRR), age, gender, body weight, treatment duration, and other clinical parameters were obtained from the Hospital medical records.

### Natalizumab Serum Concentrations

Serum concentrations of natalizumab were measured at Sanquin Laboratories (Amsterdam) by an ELISA technique, previously described (27). Briefly, the technique requires specific polyclonal rabbit anti-natalizumab F (ab) 2 fragments as capture reagent and a mouse anti-human IgG4 (anti-hIgG4) monoclonal antibody for detection.

### α4-Integrin Saturation (RO)

The RO was measured by flow cytometry with an anti-human IgG4-PE to reveal the binding of natalizumab to the surface of the lymphocytes. Three aliquots of whole peripheral blood were washed twice with phosphate-buffered saline to eliminate soluble IgG4 immunoglobulins present in the plasma of the patients to which anti-IgG4-PE could be bound. One of the aliquots was incubated with saturating natalizumab (10 μg/mL) for 1 h at 4°C temperature whereas the remaining two were kept in ice without natalizumab. After incubation, immunofluorescence was carried out against the following human molecules with the next panel of labeled antibodies: CD4-FITC, IgG4-PE, CD19-PerCP, CD3-V500, and CD8-APC-H7 (BD Biosciences, San José, CA). One of the tubes without natalizumab was used for fluorescence minus one (FMO)-PE control. The incubation was made in darkness for 30 min at 4°C. Then, red blood cells were lysed with FACS lysing solution (BD Biosciences, San José, CA) for 10 min and the remaining leukocytes were suspended with phosphate-buffered saline−1% fetal calf serum. The percentage of the different lymphoid subsets and the mean fluorescence intensity (MFI) of the PE signal were measured with a FACSCanto II flow cytometer (BD Biosciences, San José, CA) collecting 100,000 nucleated events. The percentage of natalizumab saturation was calculated from the MFI in each sample by the following formula: MFI – hIgG4-PE signal (without natalizumab)/MFI – hIgG4-PE signal (with natalizumab) × 100.

### Statistics

Sample size predetermination was focused on the association between RO and plasma levels. Assuming a minimum correlation = 0.50, an alpha risk of 0.05 and a beta risk of 0.20, we obtained a sample of 30 patients.

Variables analyzed included serum concentration of natalizumab, α4-integrin saturation (RO), weight, height, body surface area, BMI, age, gender, time to treatment start, and dose scheme [either as continuous or categoric variable (SID vs. EID)].

Descriptive analysis of the sample: quantitative variables were described by their measures of central tendency (mean) and dispersion (standard deviation); qualitative variables were described by their proportion and number of patient. Differences of mean were analyzed by *t*-test. Homoscedasticity was tested with Levened and since the *t-*test is a robust test (*n* ≥ 30), normality was not checked. Heteroscedasticity variables were analyzed with an unequal variance *t*-test. Spearman's correlation (rho) was used for association analysis of quantitative variables. A linear regression model was applied to control for possible confounding variables. A 95% CI was calculated for all estimates. A *p* < 0.05 was considered statistically significant. All analyses were performed using Stata version 13.

## Results

### Patient Demographics

The 32 patients enrolled for the study experienced at least 1 relapse in the previous 12 months before being treated with natalizumab and their characteristics are summarized in Table 1.

**Table 1:** Patient demographic and clinical characteristics.

Participants (*n*)	32
Male [*n* (%)]	15 (49%)
Female [*n* (%)]	17 (51%)
Age [Mean ± SD]	40.60 ± 8.61
Weight (kg) [Mean ± SD]	72.75 ± 14.71
Height [Mean ± SD]	170.80 ± 8.14
JC virus positive [*n* (%)]	11 (33.33%)
JC virus index [Mean ± SD]	0.47 ± 0.25
Treatment duration, years [Mean ± SD]	4.51 ± 2.68
Dose interval length, days [Mean ± SD]	33.2 ± 3.21
Days prior infusion by scheme [Mean ± SD]	*SID* 29.87 ± 1.96
	*EID* 35.47 ± 1.42
NTZ doses [Mean ± SD]	53.18 ± 31.04
Patients with prior treatment [n (%)]	
Interferon	27 (81.81%)
Glatiramer acetate	15 (45.45%)
Dimethyl fumarate	8 (24.24%)
Fampridine	3 (9.09%)
Reasons for abandonment of prior treatment	1 (3.03%)
Efficacy reasons	18 (66.6%)
Intolerability	7 (25.92%)
Lack of compliance	2 (6.06%)

*SD, standard deviation; JCV, JC virus; SID, standard interval dose; EID, extended interval dose*.

According to the Expanded Disability Status Scale (EDSS) (23), 26 patients (81.25%) presented mild to moderate impairment (EDSS ≤ 4), while 6 (18.75%) patients presented mild to moderate disability (EDSS between 4 and 7): no patient exceeded (EDSS > 7) thus unable to walk beyond ~5 m. The mean (SD) EDSS score at the start of the study was 2.599 ± 1.520 while at the end it was 2.484 ± 1.486.

No participant missed a scheduled dose of natalizumab and all patients received the full 300 mg dose. There were no major adverse events that could have prevented the administration of a dose to a patient. All patients were radiologically stable and did not relapse during the study.

### Natalizumab Serum Concentrations

The serum concentration of natalizumab was highly varied, ranging from 0.72 to 67 μg/ml: the concentration was constant over time for the same patient and most individuals. A variation in concentration of more than 15 ug/ml was observed in four patients. The rest of the patients had quite a good concordance in serum concentrations amongst samples from the same individual. However, variation among serum natalizumab concentrations between one individual and another were high, ranging from 1–2 to 50 or more μg/ml.

In view of these observations, we analyzed individual factors which could affect the PK and PD of natalizumab, including body weight, height, body surface area, BMI, age, gender, dose scheme, and duration of treatment.

**Figure 1:**
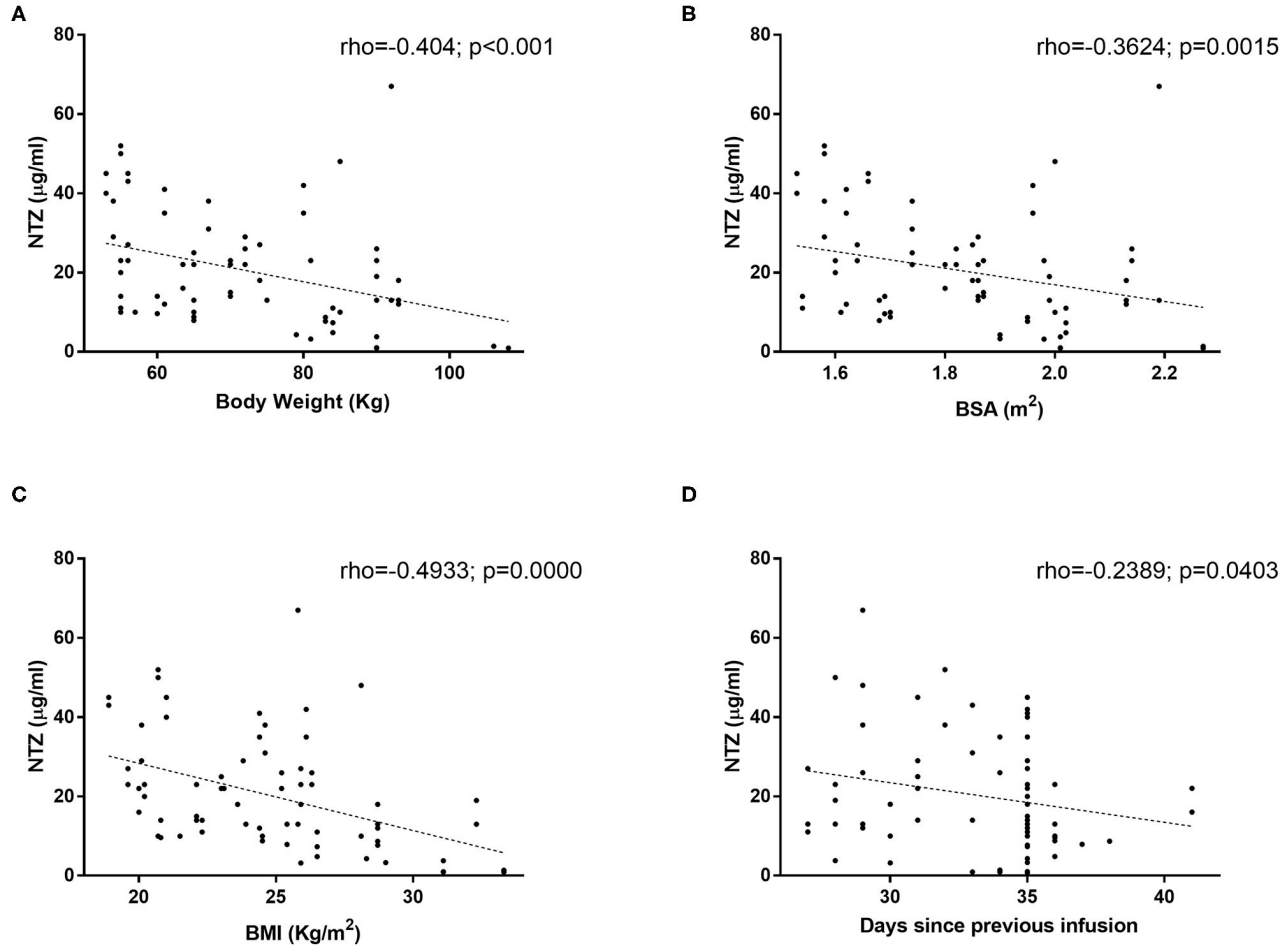
Natalizumab serum concentration correlation to **(A)** body weight, **(B)** Body Surface Area (BSA), **(C)** Body Mass Index (BMI), and **(D)** scheme doses. Natalizumab concentration was measured with ELISA as described by Rispens et al. in 74 samples from 32 patients. Spearman's correlation (rho) was used for association analysis of quantitative variables.

Among individual characteristics, we found a negative linear relationship between serum concentrations of natalizumab and patient body weight (rho = −0.404; *p* < 0.001; Figure 1A), body surface area (−0.3624; *p* = 0.0015; Figure 1B), and BMI (−0.4933; *p* = 0.0000; Figure 1C), when considered as continuous variables. Similarly, when we analyzed the correlation between serum concentration and the interval between doses in each sample we saw a slight negative correlation between post-infusion days and plasma concentration of natalizumab (rho = −0.2389; *p* = 0.0403; Figure 1D). This correlation between body weight and natalizumab concentrations was seen with similar strength in both treatment schemes (SID rho = −0.41 *p* < 0.04 vs. EID rho = −0.42 *p* < 0.002; data not shown).

Multivariate analysis confirmed that body weight, BMI and dose interval influence the serum levels of natalizumab (Tables 2, 3).

**Table 2:** Multivariate analysis for drug serum levels and body weight (variables are considered as continuous).

**Natalizumab μg/ml**	**β**	**[95% Conf. Interval]**	**P-value**
Weight (kg)	−0.3736135	−0.5905907 to 0.1566363	0.001
Age (years)	0.1156723	0.479519 to 0.2661744	0.548
Dose scheme (days)	−1.123487	−2.119463 to 0.1275116	0.028
Cons.	89.53663	52.32627 to 126.747	0.000

**Table 3:** Multivariate analysis for drug serum levels and BMI (variables are considered as continuous).

**Natalizumab μg/ml**	**β**	**[95% Conf. Interval]**	**P-value**
BMI (Kg/m2)	−1.779968	−2.597465 to −0.9624717	0.000
Age (years)	−0.0439754	−0.4149139 to 0.3269631	0.814
Dose scheme (days)	−1.192826	−2.148883 to −0.2367683	0.015
Cons.	105.8316	67.78114 to 143.882	0.000

Finally, we found a weak association between natalizumab serum concentration and patient age (*p* = 0.033; data not shown) while the duration of the treatment did not influence the serum levels of the drug (data not shown).

### α4-Integrin Receptor Occupancy

To evaluate the PD of natalizumab we measured the RO in the T-cells from the peripheral blood of patients by using flow cytometry. Just as with natalizumab concentrations we analyzed the impact of different individual characteristics as well as the scheme of treatment on the RO of natalizumab.

**Figure 2:**
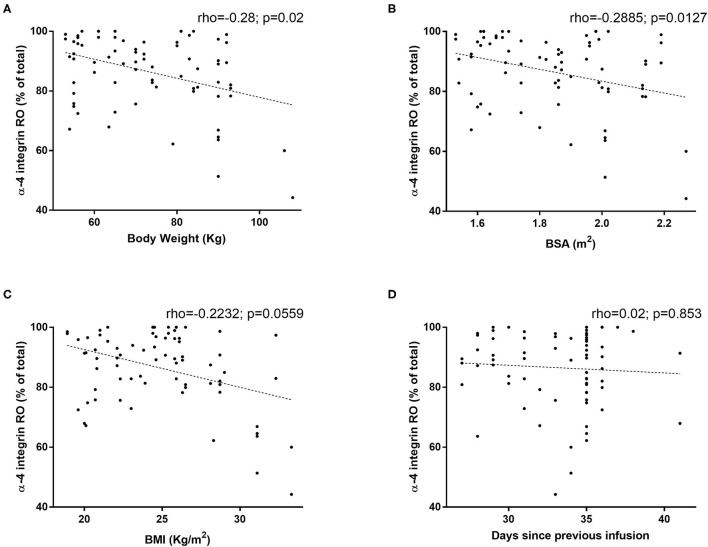
Correlation of receptor occupancy of α4-integrin by natalizumab to **(A)** body weight, **(B)** Body Surface Area (BSA), **(C)** Body Mass Index (BMI), and **(D)** scheme doses. The receptor occupancy of natalizumab was measured by flow cytometry, with an anti-human IgG4-PE, by revealing the binding of natalizumab to the surface of the lymphocytes in 74 samples from 32 patients. Spearman's correlation (rho) was used for association analysis of quantitative variables.

The evaluation of natalizumab occupancy of α4-integrin also revealed high variability among patients, ranging from 44 to 100% (data not shown). Natalizumab occupancy of α4-integrin was weakly inversely related to the body weight when the whole cohort was considered (rho = −0.28; *p* = 0.02; Figure 2A). Similar correlations were obtained with both body surface area and BMI (rho = −0.2885; *p* = 0.0127; rho = −0.2232; *p* = 0.0559; Figures 2B,C). When patients were divided on SID or EID scheme of treatment, natalizumab occupancy of α4-integrin was related to body weight only in those patients treated with the EID scheme (rho = −0.416, *p* = 0.003). Actually, most patients with values of RO below 70% were high weight individuals from the EID scheme. In addition, the treatment scheme did not influence the RO, either as a categorical (SID or EID: 88.41 vs. 85.67, *p* = 0.367) or continuous variable (rho = 0.02; *p* = 0.853; Figure 2D).

Similarly, the age, gender or treatment duration were not related to the RO neither considering patients as a whole cohort nor divided by the SID or EID scheme of treatment (data not shown). The multivariate analysis confirmed the inverse association between RO and body weight (beta = −0.31; *p* = 0.001) and BMI (beta = −1.39; *p* = 0.001; Tables 4, 5).

**Table 4:** Multivariate analysis for receptor occupancy of α4-integrin and body weight (variables are considered as continuous).

**RO (% of total)**	**β**	**[95% Conf. Interval]**	**P-value**
Weight (Kg)	−0.3124683	−0.4981626 to −0.126774	0.001
Treatment duration (years)	0.9801092	−0.0592339 to 2.019452	0.064
Cons.	104.8731	90.08744 to 119.6587	0.000

**Table 5:** Multivariate analysis for receptor occupancy of α4-integrin and BMI (variables are considered as continuous).

**RO (% of total)**	**β**	**[95% Conf. Interval]**	**P-value**
Age (years)	0.1634503	0.2065952 to 0.5334958	0.381
Dose scheme (days)	−0.5334701	−1.43738 to 0.37044	0.243
Treatment duration (years)	0.0029582	−0.1976502 to 0.2035667	0.977
BMI (Kg/m2)	−1.398437	−2.172268 to −0.6246052	0.001
Cons.	132.2103	96.08135 168.3393	0.000

### The Receptor Occupancy Depended on the Serum Concentration of Natalizumab

**Figure 3:**
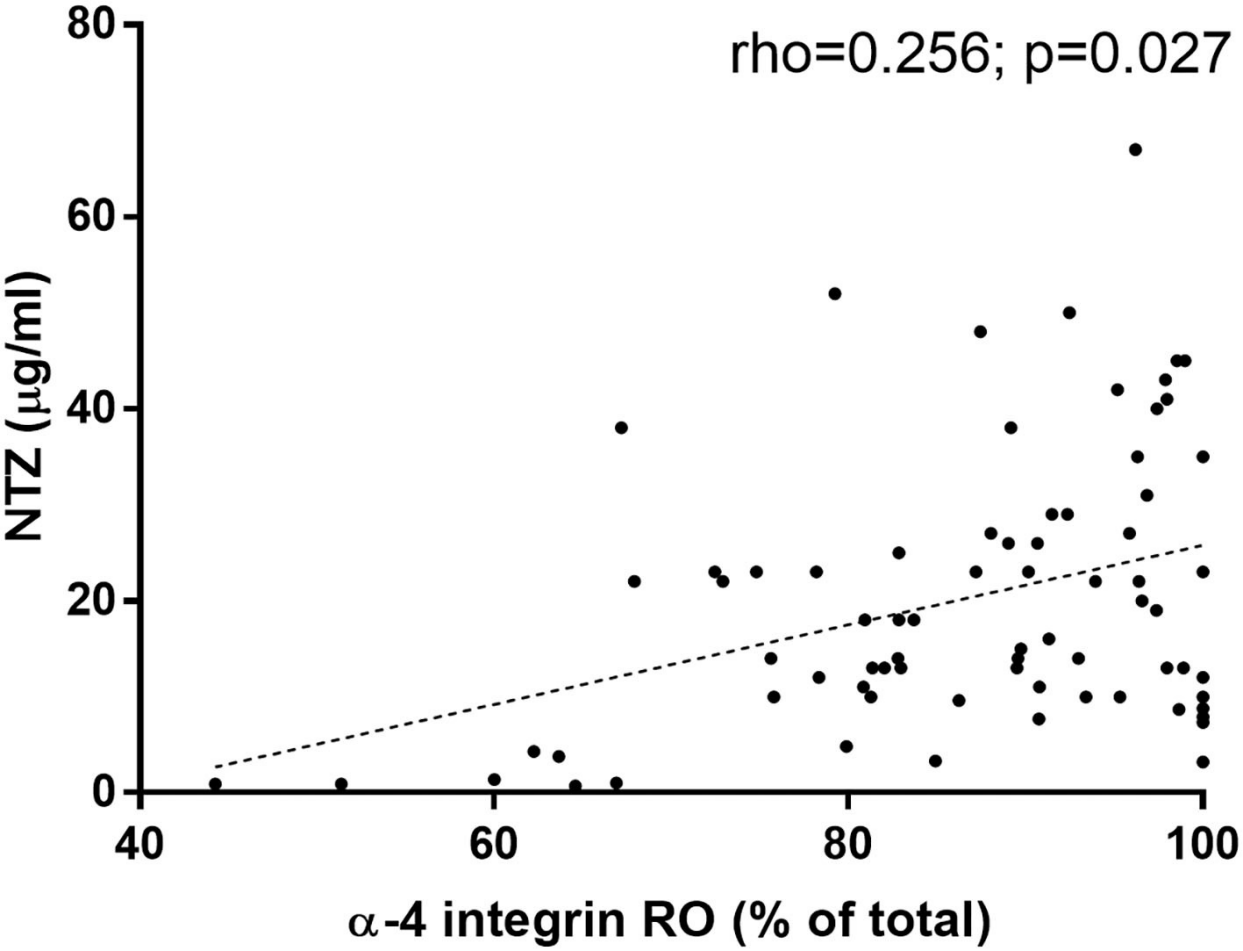
Receptor occupancy of α4-integrin by natalizumab correlates to natalizumab serum concentration. Natalizumab concentration was measured with ELISA as described by Rispens et al. The receptor occupancy of natalizumab was measured by flow cytometry with an anti-human IgG4-PE, by revealing the binding of natalizumab to the surface of the lymphocytes in 74 samples from 32 patients.

Finally, we wanted to confirm the dependence of the RO on the plasma concentration of natalizumab in our cohort of patients. A weak association was found between both parameters in the cohort of patients (rho = 0.256, *p* = 0.027; Figure 3); in the case for male patients (rho = 0.477, *p* = 0.019); in the group of patients above 40 years old (rho = 0.453, *p* = 0.04); and in the EID scheme (rho = 0.416, *p* = 0.003). A multivariate analysis confirmed that the higher the natalizumab concentration the higher the RO adjusted of gender, age and scheme of treatment (Table 6).

**Table 6:** Multivariate analysis confirming the relation between natalizumab concentration and receptor occupancy of α4-integrin.

**RO (% of total)**	**β**	**[95% Conf. Interval]**	**P-value**
Natalizumab (ug/ml)	0.2310722	0.0354729 to 0.4266714	0.021
Weight (Kg)	−0.229495	−0.4227673 to −0.0362227	0.021
Treatment duration (years)	0.9835752	−0.0242292 to 1.99138	0.056
Cons.	94.135	77.15946 to 111.1105	0.000

The weak association between RO and plasma levels of natalizumab in the whole cohort was due to the fact that for over 10 μg/ml there was no correlation between RO and plasma concentration of natalizumab (Figure 3). In the light of this, correlations of up to 10 μg/ml and above 10 μg/ml were calculated separately: the values were for up to 10 μg/ml rho = 0.58 (*p* = 0.006) and above 10 μg/ml rho = 0.23 (*p* = 0.091). Moreover, the cut-off point with the highest statistic correlation between both parameters was 9 μg/ml (rho = 0.71; *p* = 0.003).

## Discussion

Our study adds insights on how the individual characteristics of the patients which mostly influence the PK (natalizumab serum concentration) and the PD (natalizumab RO) of natalizumab in 32 effectively treated RRMS patients for a long period of time with no major adverse reactions (data given in Table 1).

The long-standing experience reached with natalizumab in clinical trials and in observational studies offers strong evidence of its effectiveness in RRMS patients (3–5). However, there has been some concern for the patient safety since natalizumab was linked to some cases of PML (6, 7). Established risk factors for PML include the level of anti JCV antibodies in serum as assessed by anti-JCV antibody index, the use of immunosuppressant therapy before natalizumab initiation, and the duration of natalizumab treatment (6, 7). Moreover, it is more than likely that the EID scheme would develop this severe disease to a lesser extent and that this scheme could effectively control the RRMS in the same way. To confirm this possibility several studies were carried out by different authors (11, 18–22, 27–29). Currently, there are two clinical trials in this field, one assessing the effect of a planned 12-week dosing interruption on the drug PK (NCT04048577), and another to evaluate the impact of switching the patients from SID to EID on the effectiveness of natalizumab (NCT04580381).

The evaluation of natalizumab serum concentrations in our patients revealed a broad range (over 60-fold) of values, showing the pharmacological variability that exists among patients. The majority of patients had concentrations of 5 μg/ml or greater, including two patients with concentrations >50 μg/ml. This means that most patients were over-treated (19, 20), since concentrations higher than 5 μg/ml were related to complete saturation of the α4-integrin in most patients. This could increase the patient's risk of developing PML (29).

Our data demonstrate that serum concentration of natalizumab is inversely related with patient's weight, in agreement with previous studies (19, 30, 31), as well as with body surface area and BMI. Therefore, these parameters should be taken into consideration when deciding the scheme for natalizumab.

Similar to other studies the treatment scheme also impacted the plasma concentration of natalizumab, that was higher in the SID scheme (19, 21, 22). The selected patients from the SID group could, therefore, switch to the EID group since no patient relapsed during the study. Several studies after natalizumab discontinuation indicate that MS disease activity is suppressed for at least 6 weeks after the last administration of the drug (12, 14). Furthermore, 2 retrospective studies have suggested that natalizumab efficacy is not compromised by EID regimens (21, 22).

When stratified by gender, natalizumab serum concentrations were lower in men, both in the whole cohort and in the different dose schedules. However, no effect of gender was observed in the multivariate analysis, suggesting that those differences were actually related to weight differences between the sexes. Treatment duration did not influence the natalizumab serum concentrations, which is in line with other study (31). These findings reopen the discussion on whether a longer course of treatment could be supported without increasing the risk of PML (32, 33).

Regarding natalizumab RO, it has always been assumed that values between 70 and 80% were necessary for optimal drug efficacy. However, different studies show that much lower levels (around 20%) would be enough to keep the patient without significant disease activity (18, 34). This is supported by our study here where we found RO values under 70% and even some under 60% in stable patients.

In the individual characteristics that affect the RO, we found that occupancy of α4-integrin is weakly inversely related to the body weight and BMI when the whole cohort was considered. The fact that most patients were beyond saturation levels may explain the low significance, in accordance with a previous study (10). The relationship of RO and body weight found in the whole cohort was only maintained in the EID cohort, this may be due to the fact that many of our samples for EID were from heavier patients. There are no significant differences in RO values when dividing patients by the scheme of treatment, in contrast with other studies whereby those were greater in SID (19, 21). This may be a reflection of our study design, in which the EID samples were collected at ~5 weeks post infusion (Table 1), while in these other studies the time was longer, around 6 weeks. This would mean that the treatment could be extended at least 1 week without significant changes in RO.

Finally, in our cohort of patients we confirmed and characterized the correlation between the RO and the plasma concentration of natalizumab. In fact, we have described that a concentration of natalizumab of 9 μg/ ml is the cut-off point where the strongest correlation between these variables: when the natalizumab concentration increases, the α4ß1-integrin saturation increases rapidly from ~40% to 80%, but the curve flattens beyond this value. In other studies this cut-off was established at 10 μg/ml (10). It appears that physicians should avoid concentrations higher than 9 μg/ml because there is no PD advantage or clinical benefits. Furthermore, we should seek lower concentrations to obtain even lower RO values since RO values below 50% have been sufficient to keep patients without radiological and clinical disease activity, either in our study or others (18, 34). Nevertheless, we are conscious that stability on MRI and lack of clinical relapses in the short term of observation are unequivocally not identical to lack of clinical progression, neurodegeneration, and axonal loss over the longer term.

In summary, with the current approved fixed dose, there is a wide pharmacological variability between patients that prescribing clinicians should be aware and take into account. Among the individual characteristics, body weight, BMI, and the treatment scheme, had a significant impact on the pharmacology of natalizumab. In addition, RO strongly depends on natalizumab serum concentrations up to ~9 μg/ml while higher concentrations result in the saturation of the integrin in most patients. As a consequence, the majority of our patients were overtreated. Conversely, testing levels may be extremely useful to prevent inadvertent underdosing of patients, specifically those in whom extended dosing is exhibited. The possibility of underdosing should dominate clinical concerns in this group of patients. To pursue these possible outcomes larger studies will be necessary.

In summary, the authors strong argue that either the RO and/or the serum concentration of natalizumab should be routinely measured in every patient, with the aim of personalizing treatment without loss of efficacy.

## Data Availability Statement

The raw data supporting the conclusions of this article will be made available by the authors, without undue reservation.

## Ethics Statement

The studies involving human participants were reviewed and approved by the Ethics Committee of La Princesa Hospital. The patients/participants provided their written informed consent to participate in this study.

## Author Contributions

JMSL-M and CM-C: conception, data collection, analysis, and writing. YP: data collection and sample analysis. VM-L: conception, data collection, and critical revision. RJ-S: sample analysis. LV-P: analysis and critical revision. DP-S: critical revision. AV: sample analysis and critical revision. TR: sample analysis and critical revision. All authors contributed to the article and approved the submitted version.

## Funding

This work was supported by a grant PI018/01163 from the Fondo de Investigaciones Sanitarias, Instituto de Investigación Carlos III, Ministerio de Sanidad y Consumo, Spain, to CM-C who also was cofinanced by FEDER funds.

## Conflict of Interest

The authors declare that the research was conducted in the absence of any commercial or financial relationships that could be construed as a potential conflict of interest.

## Publisher's Note

All claims expressed in this article are solely those of the authors and do not necessarily represent those of their affiliated organizations, or those of the publisher, the editors and the reviewers. Any product that may be evaluated in this article, or claim that may be made by its manufacturer, is not guaranteed or endorsed by the publisher.
